# Multiple myeloma presenting with high-output heart failure and improving with anti-angiogenesis therapy: two case reports and a review of the literature

**DOI:** 10.1186/1752-1947-2-229

**Published:** 2008-07-15

**Authors:** Jason Robin, Bara Fintel, Olga Pikovskaya, Charles Davidson, Jeffrey Cilley, James Flaherty

**Affiliations:** 1 Department of Medicine, Division of Cardiology, Northwestern University Feinberg School of Medicine, Chicago, IL, USA

## Abstract

**Introduction:**

Common manifestations of multiple myeloma include osteolytic lesions, cytopenias, hypercalcemia, and renal insufficiency. Patients may also exhibit heart failure which is often associated with either past therapy or cardiac amyloidosis. A less recognized mechanism is high-output heart failure. Diuretic therapy in this setting has little efficacy in treating the congested state. Furthermore, effective pharmacotherapy has not been established. We report two patients with multiple myeloma and high-output heart failure who failed diuretic therapy. The patients were given dexamethasone in conjunction with lenalidomide and thalidomide, respectively. Shortly thereafter, each patient demonstrated a significant improvement in symptoms. This is the first report of successful treatment of multiple myeloma-induced high-output failure via the utilization of these agents.

**Case presentation:**

Two patients with multiple myeloma were evaluated for volume overload. The first was a 50-year-old man with refractory disease. Magnetic resonance imaging demonstrated diffuse marrow replacement throughout the pelvis. Cardiac catheterization conveyed elevated filling pressures and a cardiac output of 15 liters/minute. He quickly decompensated and required mechanical ventilation. The second patient was a 61-year-old man recently diagnosed with multiple myeloma and volume overload. Skeletal survey demonstrated numerous lytic lesions throughout the pelvis. His cardiac catheterization also conveyed elevated filling pressures and a cardiac output of 10 liters/minute. Neither patient responded to diuretic therapy and they were subsequently started on dexamethasone plus lenalidomide and thalidomide, respectively. The first patient's brisk diuresis allowed for extubation within 48 hours after the first dose. He had a net negative fluid balance of 15 liters over 10 days. The second patient also quickly diuresed and on repeat cardiac catheterization, his cardiac output had normalized to 4.7 liters/minute.

**Conclusion:**

Multiple myeloma can cause high-output failure. The mechanism is likely extensive bony involvement causing innumerable intramedullary arteriovenous fistulas. Diuretic therapy is not effective in treating this condition. Lenalidomide and thalidomide, both of which inhibit angiogenesis, seem to be viable treatment options. Based on the rapid and effective results seen in these two patients, a potential novel mechanism of 'pharmacologic fistula ligation' with these agents may be the most effective way to treat this presentation.

## Introduction

Multiple myeloma is characterized by the neoplastic proliferation of a single clone of plasma cells producing a monoclonal immunoglobulin. The proliferation of plasma cells in the bone marrow results in extensive skeletal destruction with osteolytic lesions, osteopenia, and pathologic fractures. Other common clinical findings include cytopenias, hypercalcemia, recurrent bacterial infection and renal insufficiency. Cardiac pathology has also been well described with multiple myeloma. When new onset heart failure is seen in the setting of multiple myeloma, systemic amyloidosis with light chain deposition in the myocardium is often at the top of the differential diagnosis. Other etiologies which warrant consideration are former drug therapies as well as underlying ischemia. However, another mechanism which receives less attention is myeloma-induced high-output failure. This typically presents in patients with extensive bony involvement and the diagnosis is supported by physical exam findings, echocardiography, and cardiac catheterization. In these patients, traditional heart failure therapies such as beta blockers, ACE inhibitors and diuretics are not useful and may be detrimental. As with other causes of high-output failure such as profound anemia, thiamine deficiency, thyrotoxicosis and cirrhosis, the treatment is to correct the underlying cause of the high-output state. With multiple myeloma, there is literature which supports the high-output state being secondary to innumerable intramedullary arteriovenous fistulas [1,2]. If this is the case, pharmacotherapy with the ability to target the underlying malignancy and inhibit angiogenesis is an intriguing therapeutic option. Lenalidomide and thalidomide, both of which are acceptable therapies for multiple myeloma, have these pharmacological properties. We describe two cases of multiple myeloma associated with high-output failure that rapidly responded to the initiation of these agents.

## Case presentation

### Case 1

**Figure 1:**
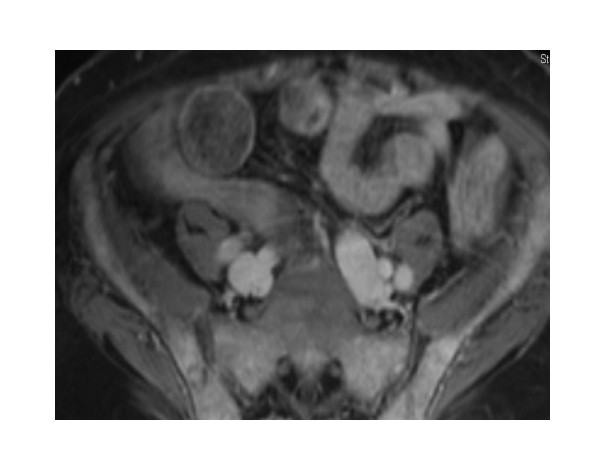
**MRI pelvis**. Diffuse bone marrow replacement throughout the pelvis and proximal femora with only small areas of residual fatty marrow in the greater trochanters and femoral heads bilaterally. The diffuse enhancement is consistent with extensive disease.

**Figure 2:**
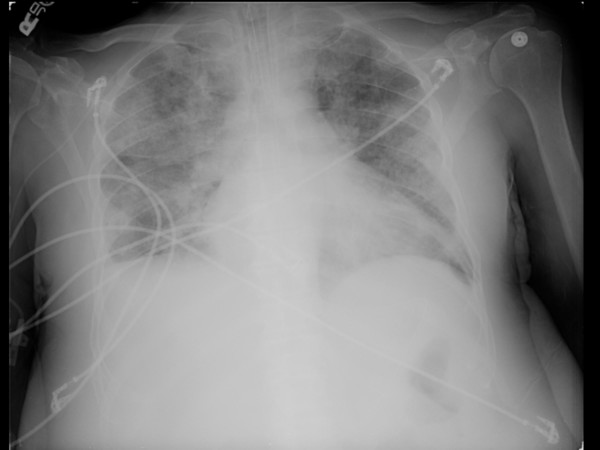
**Chest X-ray**. Cardiomegaly with diffuse bilateral interstitial infiltrates and a right-sided pleural effusion.

A 50-year-old man of Indian ancestry who was diagnosed with multiple myeloma three years earlier was evaluated in our hospital. His only other chronic medical issue was mild hypertension. His myeloma had progressed rapidly since diagnosis despite a variety of therapies over the years including systemic corticosteroids, cyclophosphamide, etoposide, cisplatin, stem cell transplantation, thalidomide, and for the most recent three months, bortezomib. Blood work and magnetic resonance imaging at a recent out-patient visit demonstrated pancytopenia as well as diffuse myelomatous bone marrow replacement throughout his pelvis and proximal femora (Figure 1). At this time, he was being hospitalized due to extensive fluid retention in the abdomen and lower extremities as well as dyspnea. He stated that he had gained 15 pounds over the past two weeks. On initial examination, he was afebrile with a heart rate of 100 beats/minute and a blood pressure of 97/50 mmHg. His oxygen saturation was 96% while receiving oxygen at 3 liters/minute by nasal cannula. He had crackles at the bases of his lungs bilaterally. His cardiovascular exam was remarkable for 12 cm of jugular venous distension and tachycardia with a 2/6 systolic flow murmur at the left upper sternal border. His abdomen was distended with shifting dullness to percussion and a liver edge 4 cm below the right costal margin. His extremities were warm to touch with 3 + bilateral lower extremity edema as well as significant scrotal edema. Pertinent initial laboratory studies were remarkable for a hemoglobin of 9.1 g/dl, a platelet count of 10,000 per microliter, a blood urea nitrogen of 55 mg/dl, a creatinine of 1.0 mg/dl, an albumin of 3.6 g/dl, and a calcium of 13 mg/dl. The ECG demonstrated sinus tachycardia with normal voltage and diffuse T wave flattening. His chest X-ray demonstrated mild cardiomegaly and evidence of pulmonary edema. An echocardiogram conveyed a hyperdynamic left ventricle with normal wall thickness, no regional wall motion abnormalities, no valvular abnormalities and normal diastolic function. Thrice daily intravenous furosemide was administered for the first ten hospital days. Despite aggressive diuretic therapy, the patient's volume status worsened. On the eleventh hospital day, cardiac catheterization was performed (Table 1). Based on the high output values obtained at catheterization, a thyroid panel was obtained which was unremarkable. In addition, he was given empiric thiamine replacement, placed on broad-spectrum antibiotics for possible sepsis, and was started on a continuous intravenous infusion of furosemide. His respiratory status continued to worsen and on hospital day number 14, he required intubation and mechanical ventilation for hypoxemic respiratory failure (Figure 2). His volume status continued to worsen over the next 2 days despite the aforementioned therapy. As a last resort, it was decided to initiate therapy targeting the underlying myeloma on hospital day 17. Lenalidomide 25 mg and dexamethasone 40 mg daily were administered through the patient's nasogastric tube. Within 24 hours, a brisk diuresis was observed and he was successfully extubated on hospital day 19. Dexamethasone was discontinued per protocol after hospital day 20, though lenalidomide was continued. By hospital day 27, he had a net negative fluid balance of 15 liters and he was discharged out of the intensive care unit. Unfortunately, on hospital day 35 in the setting of his long standing refractory thrombocytopenia, he developed a massive upper gastrointestinal bleed that could not be controlled despite aggressive resuscitory efforts and died within hours.

**Table 1:** Cardiac catheterization

	Case 1	Case 2		
	**Prior to treatment with Lenalidomide**	**Prior to treatment with Thalidomide**	**After treatment with Thalidomide**	**Normal Values**

**Heart Rate/Minute**	119	85	41	60–100
**Right Atrium (mm Hg)**	18	29	17	0–8
**Right Ventricle: Systolic/Diastolic, End Diastolic (mm Hg)**	58/3, 22	75/10, 24	57/0, 16	15–30/0–8, <12
**Pulmonary Artery (mm Hg); O_2 _Saturation**	49/15; 84%	72/22; 65%	52/15; 68%	15–30/4–12; ~70%
**Pulmonary Capillary Wedge Pressure (mm Hg)**	32	26	13	1–10
**Left Ventricle: Systolic/Diastolic, End Diastolic (mm Hg)**	86/11, 24	151/6, 25	Not Available	100–140/0–8, <12
**Aortic Pressure (mm Hg); O_2 _Saturation**	74/49; 96%	150/80; 96%	144/76; 96%	100–140/60–90; >95%
**Hemoglobin (mg/dl)**	9.4	9.1	12.9	14–16
**Cardiac Output (L/min)**	15.17	10.60	4.65	4–8
**Systemic Vascular Resistance (dynes-sec-cm^-5^)**	227	740	940	770–1500
**Pulmonary Vascular Resistance (dynes-sec-cm^-5^)**	11	166	180	20–120

### Case 2

A 61-year-old African-American man with a history of coronary artery disease presented to his internist with complaints of fatigue and lower extremity edema. On examination, he was afebrile with a heart rate of 75 beats/minute and a blood pressure of 127/70 mmHg. His oxygen saturation was 97% on room air. He had faint crackles at the bases of his lungs bilaterally. His cardiovascular exam was remarkable for 10 cm of jugular venous distension, a regular rhythm, and a 2/6 systolic flow murmur at the left upper sternal border. His abdominal examination was benign. His extremities were warm to touch with 2 + bilateral lower extremity edema. Pertinent laboratory studies were remarkable for a hemoglobin of 9.1 g/dl, a platelet count of 105,00 per microliter, a blood urea nitrogen of 13 mg/dl, a creatinine of 1.0 mg/dl, an albumin of 3.9 g/dl, and a calcium of 9.2 mg/dl. His ECG demonstrated normal sinus rhythm, normal voltage and left atrial enlargement. An echocardiogram with Doppler conveyed hyperdynamic left ventricular function with an ejection fraction of 70%, no wall motion abnormalities, mild concentric left ventricular hypertrophy, normal diastolic function, moderate to severe left atrial enlargement (47 cc/m^2^) and no valvular abnormalities. A bone marrow biopsy was performed and revealed a monoclonal population of lambda-positive plasma cells making up 90% of the total cell population. A skeletal survey demonstrated multiple lytic lesions throughout the pelvis, right humerus and skull. While the diagnosis of multiple myeloma was being investigated, the patient developed worsening lower extremity edema despite oral furosemide therapy. Cardiac catheterization was subsequently performed (Table 1). Based on the diuresis noted in the first case, it was decided to initiate thalidomide 50 mg daily and increase the dose to 200 mg over the next 4 weeks. He was also given oral dexamethasone. Two weeks after the initiation of therapy, he no longer had peripheral edema. The thalidomide/dexamethasone therapy was continued as he remained euvolemic and he was taken for a repeat cardiac catheterization two months after the initiation of therapy (Table 1). Based on his much improved clinical status, he is currently being evaluated for stem cell transplantation.

## Discussion

Volume overload in the setting of multiple myeloma is not uncommon and is usually attributed to low protein states, renal failure, amyloid-related nephrotic syndrome, or congestive heart failure. When heart failure is suspected, considerations include amyloidosis, former therapies, ischemia, and high-output failure. The pathophysiology behind myeloma-induced high-output failure is not entirely understood, but hypotheses include increased splenic flow due to splenomegaly, a plasma cell produced cytokine mediated process (IL-2, IL-6, Gamma Interferon) or perhaps innumerable, small diffuse intramedullary arteriovenous fistulas [3]. The latter seems to have the most supporting data.

In a study by McBride [4], 34 patients with multiple myeloma were evaluated. Each patient had a cardiac index calculated. Other variables evaluated included hemoglobin, calcium, quantification of the monoclonal protein, stage of disease and degree of bony involvement. When separating the cohort into those with an elevated cardiac index (>4 liters/minute/m^2^) and a normal cardiac index (<4 liters/minute/m^2^), the only variable which was statistically different between the two groups was the degree of bone involvement (p = 0.001) [4]. Thus, extensive bone involvement has the propensity to promote a high-output state.

The precise mechanism behind bone involvement promoting a high-output state was elucidated by Inanir and colleagues [2]. In their study, 11 patients with multiple myeloma and a cardiac index >4.0 liters/minute/m^2 ^were evaluated. By injecting 99mTc-macroaggregated albumin bubbles into the femoral artery as well as the antecubital vein, an arteriovenous shunting ratio was calculated by assessing the degree of pulmonary uptake after arterial and venous injection. Any degree of pulmonary uptake after arterial injection would invoke a degree of shunting because these albumin bubbles should be trapped in the first capillary bed. When comparing the cardiac indices of the 11 patients to the arteriovenous shunting ratios, there was a high correlation (coefficient, r = 0.79) which was statistically significant (p = 0.004) [2].

The management of this syndrome is challenging, and suffice to say, traditional heart failure therapy is not effective. Transcatheter embolization has been attempted in the past with temporary success [5]. Systemic chemotherapy may also be useful [1]. In our cases, we utilized systemic steroids in conjunction with the agents lenalidomide and thalidomide. Interestingly, both agents share various mechanisms of action including cytokine suppression, enhanced host immune response, and inhibition of angiogenesis. We hypothesize that the rapid improvement in heart failure after the administration of these agents may be related to each of these pharmacologic properties. However, perhaps the most relevant mechanism is the capacity to inhibit angiogenesis. Based on the proposed mechanism of high-output failure in these patients, this is an appealing and plausible hypothesis. In essence, when used in conjunction with steroids, these agents may have the ability to pharmacologically ligate intramedullary arteriovenous fistulas. Whether or not this benefit extends to patients with other etiologies of high-output failure such as Paget's disease remains to be studied.

## Conclusion

High-output heart failure is likely under-diagnosed in patients with multiple myeloma. The pathophysiology is most likely related to intramedullary arteriovenous fistulas and is most often observed in patients with extensive bone involvement. The management is not straightforward and has not been studied in large cohorts of patients. In addition, traditional heart failure therapy is unlikely to be effective. Successful management is crucial as many oncologists may be reluctant to put these patients through stem cell transplantation with the appropriate concern that the heart will not be able to tolerate the large volume shifts. Systemic steroids used in conjunction with lenalidomide and thalidomide were shown to be very successful in the management of myeloma-induced high-output failure in these two cases. We postulate that the anti-angiogenesis property of these agents may be the underlying mechanism of action which led to the dramatic improvement in volume status in these two patients. Further studies with larger numbers of patients are needed to validate these results.

## Abbreviations

ACE, Angiotensin Converting Enzyme; IL, Interleukin

## Consent

Written informed consent was obtained from both patients for publication of this case report and accompanying images. A copy of the written consent is available for review by the Editor-in-Chief of this journal.

## Competing interests

The authors declare that they have no competing interests.

## Authors' contributions

All authors were involved with the writing/reviewing and approved the final manuscript.
